# Neuronal Transcription Factors Induce Conversion of Human Glioma Cells to Neurons and Inhibit Tumorigenesis

**DOI:** 10.1371/journal.pone.0041506

**Published:** 2012-07-31

**Authors:** Junli Zhao, Hua He, Kechun Zhou, Yonggang Ren, Zixiao Shi, Zhiyuan Wu, Yizheng Wang, Yicheng Lu, Jianwei Jiao

**Affiliations:** 1 Institute of Zoology, State Key Laboratory of Reproductive Biology, Chinese Academy of Sciences, Beijing, China; 2 Institute of Neuroscience, Chinese Academy of Sciences, Shanghai, China; 3 Department of Neurosurgery, Changzheng Hospital, Second Military Medical University, Shanghai, China; 4 Ruijin Hospital, Shanghai, China; City of Hope National Medical Center and Beckman Research Institute, United States of America

## Abstract

Recent findings have demonstrated that the overexpression of lineage-specific transcription factors induces cell fate changes among diverse cell types. For example, neurons can be generated from mouse and human fibroblasts. It is well known that neurons are terminally differentiated cells that do not divide. Therefore, we consider how to induce glioma cells to become neurons by introducing transcription factors. Here, we describe the efficient generation of induced neuronal (iN) cells from glioma cells by the infection with three transcription factors: Ascl1, Brn2 and Ngn2 (ABN). iN cells expressed multiple neuronal markers and fired action potentials, similar to the properties of authentic neurons. Importantly, the proliferation of glioma cells following ABN overexpression was dramatically inhibited in both in vitro and in vivo experiments. In addition, iN cells that originated from human glioma cells did not continue to grow when they were sorted and cultured in vitro. The strategies by which glioma cells are induced to become neurons may be used to clinically study methods for inhibiting tumor growth.

## Introduction

Gliomas are the most common primary tumor of the central nervous system and are derived from the astrocytes or supportive cells in the brain [1]. There are different types of gliomas including astrocytomas, ependymomas and oligodendrogliomas. Astrocytomas, ependymomas and oligodendrogliomas arise from star-shaped astrocytes, ependymal cells lining the ventricles, and oligodendrocytes of the brain, respectively [2]. Gliomas are classified into four grades (I, II, III and IV) according to their level of malignancy [3]. Grade I gliomas are benign, slow-growing and sometimes curable by surgery. Grade II gliomas are low-grade malignant tumors and may invade the surrounding tissues. Grade III and IV gliomas are high-grade malignant tumors and are lethal within a few years. The most common and aggressive grade IV malignant glioma is Glioblastoma multiforme (GBM) [4]. Gliomas account for approximately 30% of all primary brain tumors, but they constitute 80% of tumors within the malignant subset [5]. Low grade gliomas usually exhibit a heterogeneous clinical behavior, and patients may survive 5 years or more after initial diagnosis [6]. Malignant gliomas are aggressive in nature and difficult to treat and clinical treatment only aims to improve neurological deficits and to prolong the survival time. Malignant gliomas are considered incurable and the several available approaches (including surgery, radiotherapy, and chemotherapy) only prolong the survival of most patients by a few months [7]. Therefore, it is important to investigate new approaches to treat these tumors.

Gene transfer holds promise for the treatment of malignant gliomas [8]. Previous studies have shown that overexpression of the transcription factors P53 [9], Pten [10], Pax6 [11] affect glioma growth. Although these transcription factors inhibit glioma cells proliferation, glioma cells still maintain a proliferative state. Therefore, it may be uniquely possible to convert glioma cells to other differentiated cell types such as neurons, which might further inhibit proliferation of glioma cells. Until now, it has been unclear whether glioma cells can be induced directly to become neurons, subsequently inhibiting the proliferation rate. Inspired by recent findings that fibroblasts or astrocytes can be converted to neurons by introducing defined transcription factors [12], [13], [14], we initiated our study to convert glioma cells to neurons. It was first reported that overexpression of Ascl1, Brn2 and Myt1l efficiently induces mouse fibroblasts to become functional neurons [12]. Other transcription factors, such as Ngn2 or Dlx1, are capable of converting astrocytes to neurons [14]. Cells generated by this direct conversion approach may not need to pass through the pluripotent stage, and these cells may not be tumorigenic [15].

**Figure 1:**
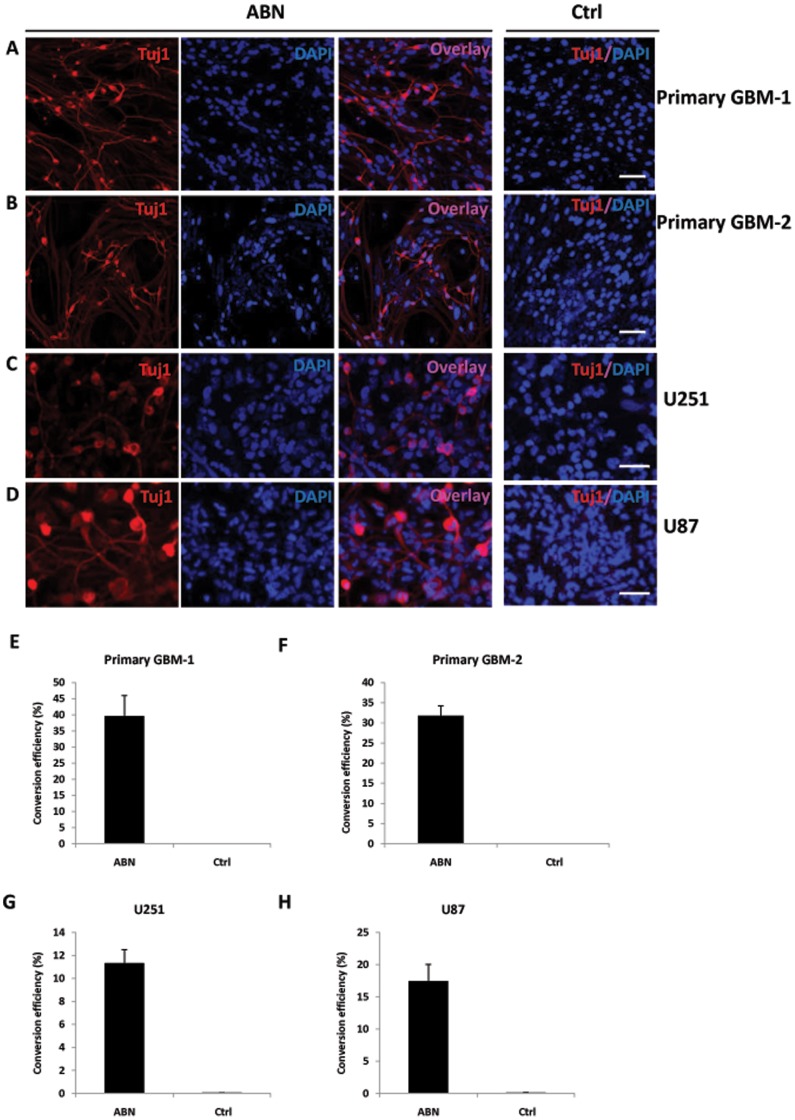
Induction of human glioma cells to neurons. A,B, Thirteen days after infection with Asc1, Brn2 and Ngn2, primary human glioma cells (GBM-1, -2) expressed neuronal protein Tuj1 and displayed a neuronal morphology. iN cell generation efficiencies estimated from Tuj1-positive cells. **C**, Induction of U251 human glioma cells to neurons by overexpressing ABN. **D**, Induction of U87 human glioma cells to neurons by overexpressing ABN. **E,F,G,H**, Quantification of iN cells induced from primary GBM-1, GBM-2, U251 and U87 cells. Scale bars: 100 µm. Error bars indicate ±s.d.

Therefore, we aimed to convert glioma cells to neurons by introducing a combination of neuronal transcription factors. In this study, we introduced transcription factors using viral infection and successfully converted glioma cells to iN cells, which displayed neuronal morphology and expressed several typical neuron-specific markers. Whole-cell patch-clamp studies also revealed that these cells exhibited both neuronal membrane properties and the ability to fire action potentials. Moreover, tumor proliferation was markedly inhibited in in vitro and in vivo experiments.

## Results

### Human Glioma Cells are Converted to Neurons by Induction with Neuronal Transcription Factors

We found that the combination of transcription factors Asc1, Brn2 and Ngn2 (ABN) effectively converts fibroblasts to neurons in previous studies [16]. Therefore, we chose this combination of transcription factors to the initiate the glioma-to-neuron induction. Genes encoding the human transcription factors Asc1, Brn2 and Ngn2 were cloned into a lentiviral vector and packaged into lentivirus particles. Human glioma cells were infected with the lentivirus particles for 4 hours and then cultured in neuron culture medium (Figure S1A). At 2 days after infection, the infection efficiency was estimated about 50∼70% for single factor and 80–90% for the combination of three factors. To rigorously exclude preexisting neurons from our initial glioma cells, the cell population was screened for the neuronal marker Tuj1; no preexisting neurons were detected (Figure S1B). Immunostaining was first performed thirteen days after infection to determine whether human glioma cells were converted to neurons. Tuj1-positive neurons were rarely observed in the control cells infected by control virus (Figure 1A,B,C,D). In contrast, 39% or 31% Tuj1-positive neurons were obtained from primary glioma cells after infection with the ABN transcription factor combination (Figure 1A,B). Two glioma cell lines, U251 and U87, were also converted to neurons with high efficiency (Figure 1C,D). We also performed the conversion by single transcription factor. Only Ascl1 acting as single transcription factor resulted in conversion of a few glioma cells to glioma cells to neurons, however the efficiency is low (less than 1%).

**Figure 2:**
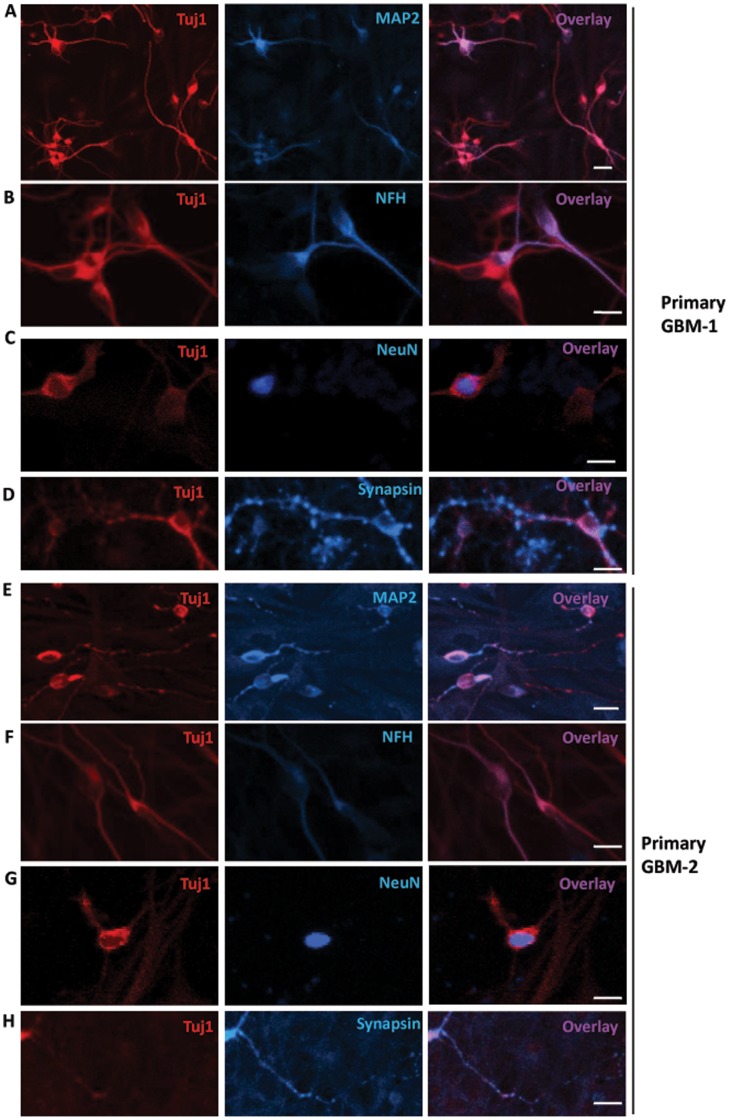
Characterization of iN cells converted from human glioma cells. **A**, GBM-1 primary human glioma cells were effectively induced to become neurons and expressed the neuronal proteins Tuj1 and MAP2 thirteen days after infection with ABN. **B,C,D**, iN cells expressed the mature neuronal proteins Neurofilament, NeuN and Synapsin three weeks after infection with ABN. **E,F,G,H**, GBM-2 primary human glioma cells expressed the neuronal proteins Tuj1, MAP2, Neurofilament, NeuN and Synapsin. Scale bars: 25 µm.

To understand the induction process in detail, infected cells were observed continuously from 2 to 40 days. Neuron-like cells bearing short, thin processes first appeared four days after infection (Figure S1C). Acquisition of neuronal morphology by iN cells coincided with the detection of Tuj1 neuronal marker expression. Similar to the development of neurons, iN cells extended longer neurite processes and gradually became more morphologically complex (Figure S1D). To exclude the effect of stem cells on glioma cells, the CD133-negative population was enriched by MACS by removing the CD133-positive cells. Following the same induction procedure, CD133-negative glioma cells were also induced to become neurons (Figure S2A,B). These experiments clearly demonstrate that glioma cells can be efficiently converted to neurons by overexpression of neuronal transcription factors.

### Characterization of iN Cells Generated from Glioma Cells

Thirteen days after induction, iN cells that originated from primary GBM1 cells showed neuronal morphology and expressed the pan-neuronal markers Tuj1, Map2, Neurofilament and NeuN (Figure 2A,B,C). The iN cells also expressed synapsin, but the number of puncta detected by immunostaining was not very high (Figure 2D). However, there was no strong expression of vGlu and GABA (not shown), even though the cells were cultured for a long period of time, suggesting that iN cells did not completely mature and release transmitters of subtype-specific neurons. Similarly, iN cells were successfully induced from primary GBM-2 cells and expressed the neuronal markers Tuj1, Map2, Neurofilament, NeuN and synapsin (Figure 2E,F,G,H).

**Figure 3:**
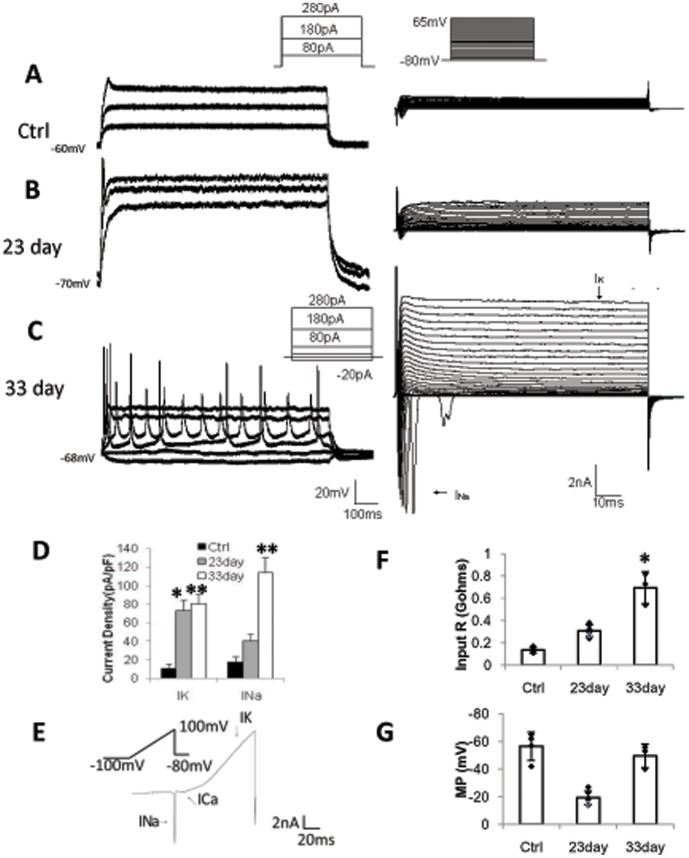
Physiological characterization of iN cells from primary human glioma cells. **A**, Representative traces of action potentials (left) and voltage dependent membrane currents (right) in control glioma cells. **B**, Representative traces of action potentials (left) and voltage gated membrane currents (right) in iN cells at 23 days after infection. **C**, Representative traces of action potentials (left) and voltage gated membrane currents (right) in iN cells at 33 days after infection. **D**, Quantification of currents density of different groups (control, n = 4; iN, 23 days, n = 6, 33 days, n = 3). **E**, Representative traces of currents measured by ramp protocol. **F,G**, Quantification of the input resistant (control, n = 4; iN, 23 days, n = 6, 33 days, n = 3), and membrane potentials (control, n = 4; iN, 23 days, n = 6, 33 days, n = 3). *:p<0.05, **:p<0.01 vs control group. Error bars indicate ±s.d. Scale bars: 25 µm.

**Figure 4:**
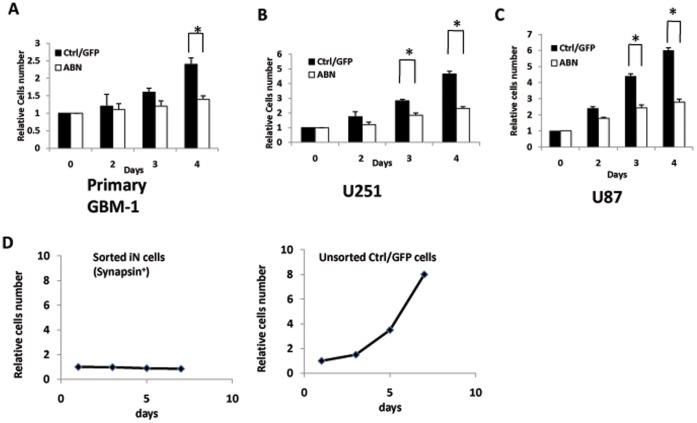
The ABN neuronal transcription factors inhibit glioma cell proliferation. **A**, Primary glioma cells were counted 2, 3 or 4 days post-infection by ABN or control virus. Cell numbers were normalized to the number of cells plated at day 0. **B**, U251 cells were counted 2, 3 or 4 days post-infection by ABN or control virus. **C**, U87 cells were counted 2, 3 or 4 days post-infection by ABN or control virus. The data represent six independent experiments. **D**, Synpasin positive cells were isolated from synapsin-mcherry infected primary human glioma cells (GBM-1). Synapsin positive cells were sorted out and cultured for 1, 3, 5 or 7 days. Synapsin positive iN cells did not grow completely. *P<0.05. Data are presented as mean ±s.d.

We next asked whether iN cells acquired neuronal properties. At 23 and 33 days after induction, the GFP-positive primary glioma cells that had an apical dendrite were chosen for analysis, and non-induced glioma cells were used as control. At 23 days after infection, the iN cells exhibited a single spike (Figure 3A,B), moreover at 33 days, iN cells fired repetitive action potentials with regular spiking pattern (Figure 3C). The majority of iN cells (9 of 11 cells) displayed conspicuous tetrodotoxin (TTX)-sensitive sodium currents and out ward potassium currents (Figure 3A,B and Figure S3). Generally, the density of the voltage-dependent currents increased gradually. Specifically, the sodium currents increased to 40±13.2 pA/pF from 18±7.3 pA/pF at day 23, and they further increased to 112±22.8 pA/pF at day 33. The potassium currents density also jumped to 71±20.2 pA/pF at day 23, and then slightly increased to 81.2±13.9 pA/pF at day 33 (Figure 3D). It should be noted that the ramp protocol used revealed a small voltage-gated calcium channel-liked conductance at day 33 (Figure 3E), which is similar to the currents recorded in converted neurons from fibroblasts [12]. In addition, the input resistance also increased gradually (Figure 3F), and membrane potentials decreased to 16.5±3.2 mV from −58.6±1.6 mV at day 23 but then recovered to 46.7±6.7 mV at day 33 (Figure 3G). These results showed that iN cells generated from glioma cells gradually acquired neuronal properties.

### ABN Transcription Factors Inhibit Glioma Cells Proliferation During the Conversion Period

Primary glioma cells, U251 or U87 cells were infected with Ascl1, Brn2 and Ngn2 lentiviral virus. For short-term cell proliferation analysis, cell growth was evaluated at 2, 3 and 4 days after infection (Figure 4A,B,C). Cells infected with the ABN lentivirus grew slowly during the first 2 days in all three cell types. Growth was markedly inhibited 3 or 4 days after ABN lentiviral infection compared with control lentiviral infection (41%, 53% and 64% inhibition at 4 days after infection with ABN virus of primary glioma cells, U251, and U87 cells, respectively).

**Figure 5:**
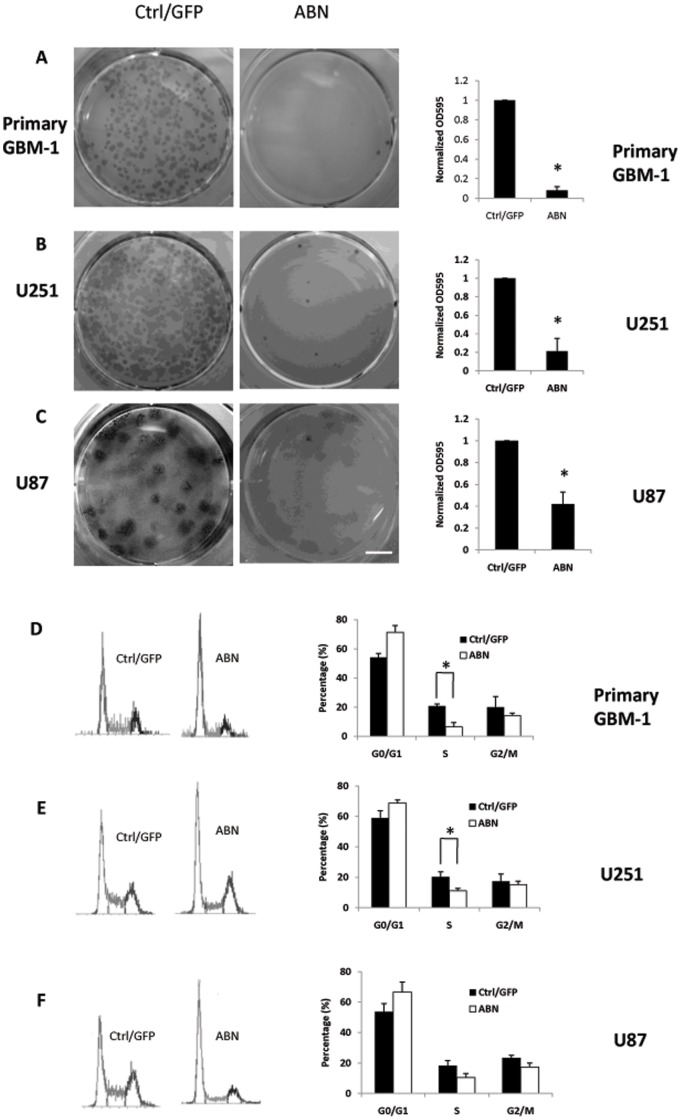
ABN neuronal transcription factors inhibit glioma cells colony formation after long-term culturing and caused arrest of the cell cycle at G0/G1. **A**, Primary glioma cells were infected with ABN and control virus. Three days after infection, cells were plated at a low density and cultured for 2 weeks. Colonies were visualized by crystal violet staining, and the density was measured by OD595. **B**, U251 cells were infected with ABN and control virus. **C**, U87 cells were infected with ABN and control virus. The data represent six independent experiments. **D**, Cell cycle distributions of primary glioma cells were analyzed three days after infection with ABN. Cells were incubated with propidium iodide at a final concentration of 10 g/ml. After incubation at room temperature for 1 h, cells were analyzed by FACS for cell cycle distribution. **E**, The U251 cell cycle distribution was detected and analyzed. **F**, The U87 cell cycle distribution was detected and analyzed. *P<0.05. Data are presented as mean ±s.d. Scale bars: 1 mm.

To determine whether the growth of iN cells was inhibited, Synapsin positive (mcherry expression) iN cells were sorted from Synapsin-mcherry (mcherry driven by synapsin promoter) and ABN infected cell. Synapsin positive iN cell did not grow 1, 3, 5 and 7 days after replating, however the unsorted control/GFP cells grew quickly (Figure 4D). The data indicated that human glioma cells completely lost their ability to proliferate after they were induced into iN cells. Neuronal transcription factors also inhibited cells growth, but they could not block the cells growth completely.

**Figure 6:**
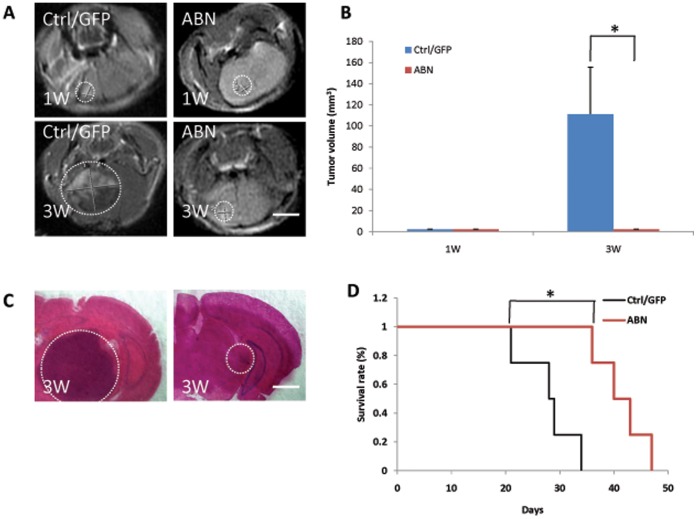
ABN neuronal transcription factors prolong mouse survival after intracranial glioma cell transplantation. **A**, Nude mice were intracranially transplanted with 5×10^5^ glioma cells infected with ABN or control virus. Representative tumor images were obtained by MRI 1 or 3 weeks after glioma transplantation. **B**, Tumor size was quantified and analyzed. **C**, Brain sections were collected and performed by HE staining. **D**, Survival of nude mice (n  = 6 mice for each condition) was evaluated by Kaplan-Meier analysis. Mice bearing gliomas from ABN-treated glioma cells survived longer than those from control-treated cells. *P<0.05. Error bars indicate ±s.d. Scale bars: 2 mm (6A), 900 µm (6C).

For long-term proliferation analysis, primary glioma, U251 or U87 cells were seeded at a low density after infection with ABN or control virus, and colonies were counted after 2 weeks to evaluate cell growth. ABN neuronal transcription factors greatly inhibited colony formation, as reflected by the number and size of the colonies that formed (Figure 5A,B,C, Figure S4A). The OD595 value was measured of the colonies for each cell type, and ABN-infected primary glioma, U251 and U87 cells showed markedly decreased OD595 values compared to control cells (Figure 5A,B,C). We also performed MTT assay to detect whether ABN inhibit glioma cell proliferation. ABN inhibited cells growth by MTT assay as well (Figure S4B). These results suggest that ABN neuronal transcription factors inhibit cell growth in primary glioma, U251 and U87 cells.

To test whether neuronal transcription factors ABN induced cell death in human glioma cells. Human glioma cells were analyzed 3 and 7 days after infection (Figure S4C). There is no significant difference (1.7%, 2.1% for control and ABN, respectively) in cell death 3 days after infection. However, ABN increased cell death after 7 days (2.8%, 6.9% for control and ABN, respectively). These results suggested that ABN may cause glioma cells death.

### ABN Leads to G0/G1 Cell Cycle Arrest

Cell cycle analysis was performed by propidium iodide staining and flow cytometry. Cell cycle assays showed that the percentage of G0/G1-phase cells increased and that of S-phase cells decreased 3 days after infection with the ABN lentivirus (Figure 5D,E,F). Control lentivirus-infected primary glioma, U251 and U87 cells showed 54.1%, 58.8%, 53.6% in the G0/G1 phase; 20.3%, 20.8%, 18.2% in the S phase and 19.9%, 17.2%, 23.3% in the G2/M phase, respectively. However, ABN-treated cells showed 71.5%, 68.7%, 66.6% in the G0/G1 phase; 6.5%, 11.2%, 10.4% in S phase and 14.2%, 15.1%, 17.2% in the G2/M phase. Furthermore, the percentage of ABN-infected cells in G0/G1 increased and those in G2/M decreased as the post-infection time increased (not shown). These results demonstrate that ABN neuronal transcription factors cause the cell cycle arrest in the G0/G1 phase.

### ABN Inhibits the Growth of Intracranially Implanted Glioma Cells in Nude Mice

To determine whether ABN decreases the tumorigenicity of glioma cells in vivo, we established an intracranial xenograft glioma nude mouse model. Five-hundred thousand control- or ABN-infected glioma cells were implanted into the brains of nude mice, after which the tumor size was evaluated by MRI and survival was monitored in both groups. The implantation experiments showed that ABN-infected glioma cells demonstrated significantly decreased tumor growth compared with the control-infected cells (Figure 6A,B). Brain sections were also collected and done with HE staining (Figure 6C). The data showed that tumor size from ABN-infected glioma cells was smaller than that from control-infected cells. Mice bearing intracranial xenografts derived from ABN-infected cells survived significantly longer than those from control-infected cells (Figure 6D). These results suggest that ABN suppresses the growth of xenografted human gliomas in a mouse model.

## Discussion

Recent studies have shown that mouse and human fibroblasts can be effectively converted to neurons [12], [13], [17]. Disease-specific fibroblasts from Alzheimer’s patients can also be converted to neurons [18]. However, it is unknown whether human glioma cells can be induced to become neurons or whether this would result in reduced proliferation rate. In this study, we showed that a combination of three transcription factors Ascl1, Brn2 and Ngn2, could efficiently convert human glioma cells to functional neurons. Similar to functional neurons, ABN iN cells derived from glioma cells expressed multiple neuronal markers and voltage-gated functional membrane channel proteins, as well as fired action potentials. During the conversion process, the proliferation of human glioma cells was greatly suppressed both in vitro and in vivo. Further the sorted iN cells did not grow over time if they were successfully induced and obtained from ABN infected human glioma cells.

Human glioma cells have relatively positive membrane potential (−50 to −10 mV), which is a property of actively proliferating cells [19], [20]. A high chloride-channel expression level is correlated with malignancy of high grade glioma, and it plays critical role in glioma migration and proliferation [21], [22]. In our study, the control glioma cells had a mean membrane potential of −58.6±1.58 mV (n = 4), and expressed chloride channel-like conductance. At an early stage of induction (23 days), although the sodium currents density was too low to fire action potentials, the changes of the membrane intrinsic properties (input resistant and membrane potential) and the expression of multiple neuronal makers (Tuj1, Map2, Neurofilament, NeuN and synapsin) indicated that glioma cells were truly altered to another type of cells. Further, at later stage of induction (33 days), the sodium currents density nearly tripled to burst repetitious action potentials. Thus, the combination of three transcription factors Ascl1, Brn2 and Ngn2 indeed drove human glioma cells to become neurons from mitotic cells.

The major challenge in our glioma cell conversion system was the possibility of neurons or neural progenitors being present in the starting material. However we did not detect neurons in our starting glioma cells when screening for neuronal markers. Furthermore, we did not detect neurons after prolonged culture of glioma cells with neuron medium. To carefully exclude the presence of neural progenitors in our glioma cells, CD133-negative cells population was sorted and used as the starting cell population for the induction and the result showed there was similar conversion efficiency with unsorted cells. These results suggested that neurons were induced directly from glioma cells with high efficiency.

It should be noted that the iN cells conversion efficiency is higher (20–40%) from primary human glioma cells than that of iN cells from primary human fibroblasts (less than 5%) in other studies [13]. The induction time from human glioma cells is short (less than 2 weeks) whereas the induction process from human fibroblasts to neurons takes longer. These results indicate that glioma cells might maintain more active transcriptional networks and are more easily induced to become neurons. Future studies should focus on increasing the induction efficiency and optimizing the culture conditions for neuronal maturation.

Although several studies have shown that neuronal transcription factors inhibit glioma proliferation [11], [23], it is not known whether transcription factors induce glioma cells to become neurons to dramatically affect cell growth. In the proliferation studies, ABN-transfected glioma cells grew slowly and formed fewer colonies. Importantly, we observed that the iN cells from human glioma cells did not grow completely. Additionally, if the glioma cells were not completely induced to neurons, ABN neuronal transcription factors inhibited glioma cell growth rate (Figure S5). After analyzing cell cycle, it seemed that glioma cells were arrested at G0/G1 phase after infection with ABN. Moreover, ABN neuronal transcription factors inhibited glioma cells growth and prolonged nude mouse survival. Future studies will be necessary to determine whether there is a difference of conversion between different grades of glioma. It will be interesting to determine whether glioma cells can be converted to neurons by direct injection of lentiviral expression viruses in vivo in an effort to reduce tumorigenicity. Although the conversion efficiency from primary human glioma cells to neurons is high (around 20–40%), it should be noted there are remaining un-converted glioma cells. The ultimate goal would be to convert all glioma cells to neurons. Therefore, further studies will be needed to increase the conversion efficiency. Also, it remains to be determined whether the converted neurons would cause side-effect, including interfering with the neural circuitry and function in the brain, or connecting to wrong target cells.

In summary, our studies indicate that human glioma cells can be induced to become neurons (iN cells), and that iN cells express multiple neuronal specific proteins and fired action potentials. During the glioma-to-neuron conversion process, the proliferation rate of glioma cells is inhibited and iN cells that originated from glioma cells could not proliferate over time. This induction approach from glioma cells to neurons may provide clues developing targeted strategies to treat gliomas.

## Materials and Methods

### Human Glioma Tissues

Human glioma tissues were collected by the Changzheng Hospital. Tumor samples were graded according to World Health Organization (WHO) criteria. Written consent was obtained from patients, and the use of human tissues was approved by the ethics committee of Changzheng hospital, Second Military Medical University.

### Cell Culture

Freshly obtained glioma tissues were first digested with 0.25% trypsin (Invitrogen) for 8 minutes and then filtered with a 70-µm cell strainer. The dissociated cells were transferred into a 60-mm culture dish with DMEM (Invitrogen) and 10% FBS (Hyclone) and cultured at 37°C in a 5% CO_2_ incubator. Every 2 days, the cells were passaged using a 1∶3 dilution. U251 and U87 glioma cell lines were cultured in the same culture medium as that used for primary glioma cells [24].

### Lentiviral Production and Viral Infection

Lentiviruses were produced from 293T cells according to a previously described protocol [25]. Briefly, genes encoding the human transcription factors Ascl1, Brn2, Ngn2 or Mytl1 were cloned into the pLenti7.3 (Invitrogen)-based lentiviral vector, which were then transfected into 293T cells together with Gag/Pol and VSV-G vectors. The culture medium was changed 12 hours after transfection, and the virus supernatant was collected at 48 hours and 72 hours after transfection. Glioma cells were infected with lentiviruses for 4 hours. After infection, the culture medium were changed to neuron medium with DMEM/Neural Basal = 1∶1, L-glutamine (2 mM), 1×B−27 and 5 ng/ml BDNF. Half of the culture medium was replaced every 2–3 days until the cells were ready for further experiments.

### Immunostaining

Immunostaining was carried out by 4% PFA fixation, 0.1% Triton permeabilization, and 5% BSA blocking [26]. The cells were then incubated with primary antibodies overnight at 4°C and secondary antibodies for 1 h at room temperature. The rabbit anti-Tuj1 (1∶1,000, Sigma), mouse anti-MAP2 (1∶500, Millipore), rat anti-Neurofilament (1∶500, Millipore), mouse anti-NeuN (1∶200, Millipore), and mouse anti-synapsin (1∶1,000, Synaptic Systems) primary antibodies were used. Alexa Fluor 488- and 546-conjugated (Invitrogen) and Cy5-conjugated (Jackson ImmunoResearch) secondary antibodies were used.

### iN Cells Efficiency Calculation

iN cells were defined as Tuj1-positive cells when they exhibit a neuronal morphology with a circular, three-dimensional appearance that extended a thin process at least three times longer than their cell body. The iN cells number was quantified at least 20 randomly selected 20×visual fields and the total Tuj1 positive cells number was calculated according to a previously described method [12]. Quantification of iN cells induction efficiency was determined by dividing the total number of iN cells by the number of total cells plated before infection.

### Cell Proliferation Assay

Glioma cells (2×10^4^) were infected with lentiviruses for 4 hours, after which the culture medium was changed to the normal glioma cell culture medium. The cells were counted 2, 3 and 4 days after infection [27]. Human glioma cells were infected with synapsin-mcherry (Mcherry driven under the synapsin promoter) together with transcription factors viruses. Synapsin-positive cells were sorted 17 days after infection by FACS (Becton-Dickinson). Synapsin-positive cells were replated and counted on day 1, 3, 5 and 7.

For colony formation experiments, glioma cells (2×10^3^) were seeded into each well of a 6-well plate 3 days after infection. After 2 weeks, the colonies were stained with 0.005% crystal violet and colony numbers were determined by measuring the OD595 of the collected cells after digestion [28].

For cell proliferation as assessed by MTT (3-[4,5-dimethythiazol-2-yl]-2,5-diphenyltetrazolium bromide; Sigma), 1500 cells were seeded into each well of 96-well-plate, cells were infected with lentiviruses for 4 hours next day. And then cells were changed to normal glioma cells medium after infection. The cell growth was performed by MTT assay 72 hours later using MTT Cell Proliferation kit (Invitrogen).

### Cell Death Assay

Human glioma cells were infected with control and ABN viruses. After 3 and 7 days, cell death was analyzed with Calcein-AM and EthD-1 staining by using Cell Live/Dead Kit (Invitrogen) according to manufacturer’s protocol.

### Cell Cycle Analysis

Glioma cells were collected 3 days after lentiviral infection. The cells were then fixed with 75% ethanol overnight at 4°C, washed three times with PBS, and incubated with 10 µg/ml Propidium Iodide and 0.2 mg/ml RNase A (Sigma) in PBS for 1 h at room temperature. The cells were then processed through a FACSCalibur flow cytometer (Becton-Dickinson), and the cell cycle distributions were analyzed by CellQuest Pro software (Becton-Dickinson) [10].

### Electrophysiology

The whole-cell configuration of the patch-clamp technique was carried out at room temperature using an Axopatch 700 A amplifier (Molecular Devices) with PCLAMP 9 software (Molecular Devices) in voltage- or current-clamp configuration. Cell capacitance (Cm) was measured by applying a 5 mV hyperpolarization step to the cell for 10 ms and calculating the area under the curve. The pipette internal solution contained 140 mM KCl 10 mM HEPES,1 mM MgCl_2_, 10 mM EGTA, 1 mM NaCl, 5 mM phosphocreatine-Tris, and 0.3 mM Na_2_ATP (pH 7.2). The bath solution contained 140 mM NaCl, 10 mM glucose, 10 mM HEPES, 1 mM MgCl_2_, and 1 mM CaCl_2_ (pH 7.35).

For current-clamp experiments, cells were held at membrane potential (around −65 to −70 mV), and step currents were injected to elicit an action potential. For voltage dependent current recording, cells were held at −70 mV, and a series of depolarizing 5 mV voltage step from −70 to +65 mV was applied every 2 s. For sodium channel blocking experiment, 200 nM TTX was used. Analyses were carried out using Origin (Microcal) and clampfit9 (Axon Instruments).

### Intracranial Tumor Growth

In the intracranial glioma model, glioma cells (500,000) infected with ABN or control viruses were intracerebrally injected into the right side (bregma: 1 mm; lateral: 2 mm; ventral: 3 mm) of the brains of nude mice [28]. Three weeks after tumor cell transplantation, mouse brains were scanned to detect tumor formation and size by MRI using a 3.0 T scanner (GE Signa HD MRI Systems). After tail vein injection of gadopentetate dimeglumine (Gd DTPA, 0.1 mmol/kg body weight), both T1- and T2-weighted images were obtained to capture the entire mouse brain [29]. Brain sections were also collected and performed with HE (hematoxylin and eosin) staining. All mouse experiments were performed according to institutional regulations.

## Supporting Information

Figure S1 **Induction of human glioma cells to neurons by infection with lentiviruses carrying transcription factors linked with IRES-GFP.** **A**, Experimental scheme for the induction of human glioma cells to neurons. Glioma cells were infected with lentiviruses for 4 h, and the medium was then changed to neuron medium. Immunostaining or physiological experiments were performed 4–30 days after induction. **B**, Our initial isolated primary human glioma cells were negative for Tuj1 staining. **C**,**D**, iN cells from primary glioma cells were positive for Tuj1 staining 4 or 13 days after infection with ABN lentivirus. iN cell morphology became more complex with longer incubation. Scale bar: 30 µm.
(TIF)

Figure S2 **CD133-negative cells sorted from primary human glioma cells are induced to become iN cells.** **A**, iN cells induced from the CD133-negative population expressed the pan-neuronal marker Tuj1. **B**, Quantification of iN cell number from the CD133-negative population and unsorted glioma cells at 13 days after infection with ABN. Scale bar: 100 µm. Error bars indicate ±s.d.
(TIF)

Figure S3 **Additional information on characterization of conversion of human glioma cells to neurons.** Voltage dependent inward sodium currents of iN cells can be totally blocked by TTX.
(TIF)

Figure S4 **Cell proliferation and cell death assay.** **A**, Colonies formed two weeks after infection with the ABN or control viruses. Human glioma cells formed smaller colonies after infection with ABN compared with the control virus. **B**, Human glioma cells proliferation was assessed by MTT assay. **C**, Cell death was performed 3 and 7 days after infection. *P<0.05. Error bars indicate ±s.d. Scale bar: 250 µm (3A), 30 µm (3C).
(TIF)

Figure S5 **A model of the effect of ABN neuronal transcription factors on glioma cells conversion and proliferation.**
(TIF)
