# Supplementary figures and images for: Neuronal Transcription Factors Induce Conversion of Human Glioma Cells to Neurons and Inhibit Tumorigenesis
Source: PLoS One. 2012 Jul 31;7(7):e41506. doi: 10.1371/journal.pone.0041506 (PMC3409237; doi:10.1371/journal.pone.0041506)

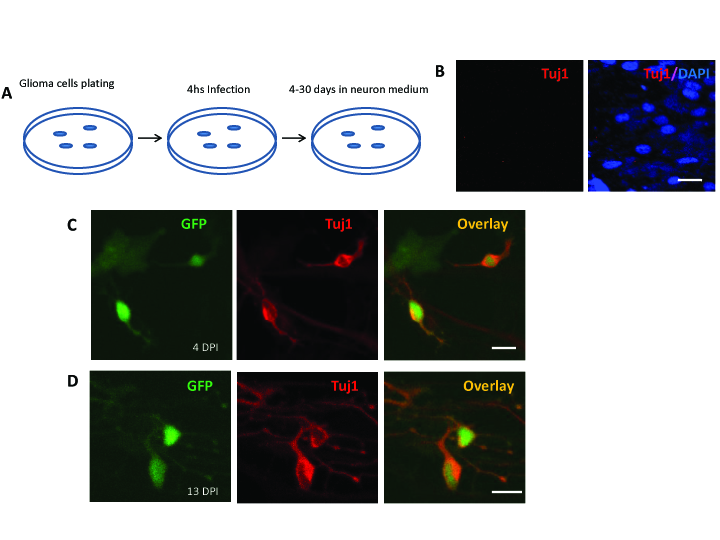

Supplement: Figure S1 — Induction of human glioma cells to neurons by infection with lentiviruses carrying transcription factors linked with IRES-GFP. A, Experimental scheme for the induction of human glioma cells to neurons. Glioma cells were infected with lentiviruses for 4 h, and the medium was then changed to neuron medium. Immunostaining or physiological experiments were performed 4–30 days after induction. B, Our initial isolated primary human glioma cells were negative for Tuj1 staining. C,D, iN cells from primary glioma cells were positive for Tuj1 staining 4 or 13 days after infection with ABN lentivirus. iN cell morphology became more complex with longer incubation. Scale bar: 30 µm. (TIF) [file pone.0041506.s001.tif]

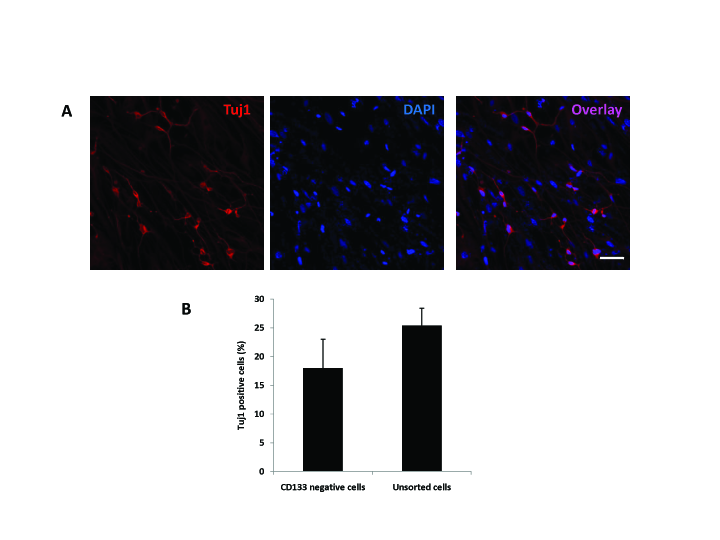

Supplement: Figure S2 — CD133-negative cells sorted from primary human glioma cells are induced to become iN cells. A, iN cells induced from the CD133-negative population expressed the pan-neuronal marker Tuj1. B, Quantification of iN cell number from the CD133-negative population and unsorted glioma cells at 13 days after infection with ABN. Scale bar: 100 µm. Error bars indicate ±s.d. (TIF) [file pone.0041506.s002.tif]

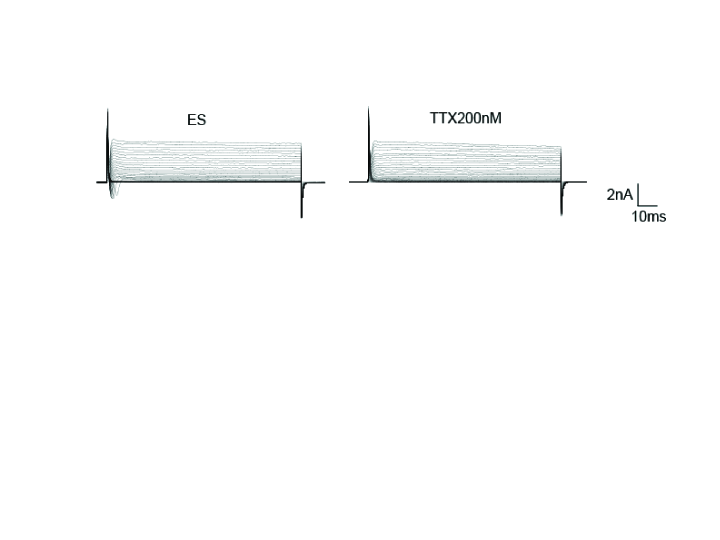

Supplement: Figure S3 — Additional information on characterization of conversion of human glioma cells to neurons. Voltage dependent inward sodium currents of iN cells can be totally blocked by TTX. (TIF) [file pone.0041506.s003.tif]

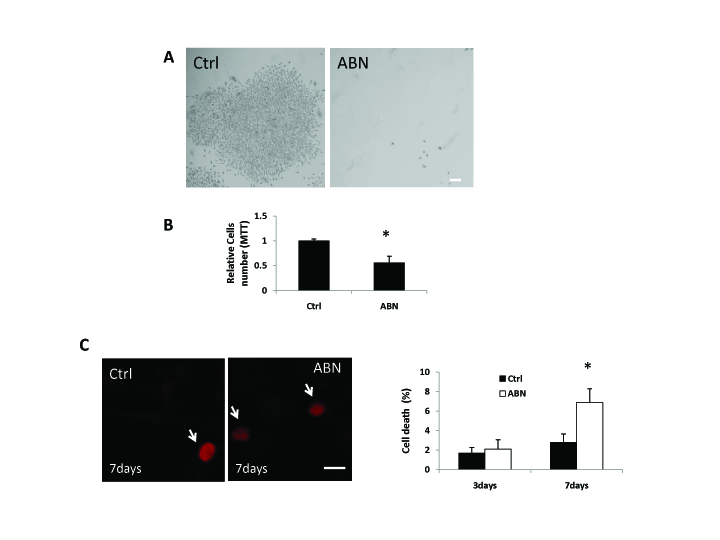

Supplement: Figure S4 — Cell proliferation and cell death assay. A, Colonies formed two weeks after infection with the ABN or control viruses. Human glioma cells formed smaller colonies after infection with ABN compared with the control virus. B, Human glioma cells proliferation was assessed by MTT assay. C, Cell death was performed 3 and 7 days after infection. *P<0.05. Error bars indicate ±s.d. Scale bar: 250 µm (3A), 30 µm (3C). (TIF) [file pone.0041506.s004.tif]

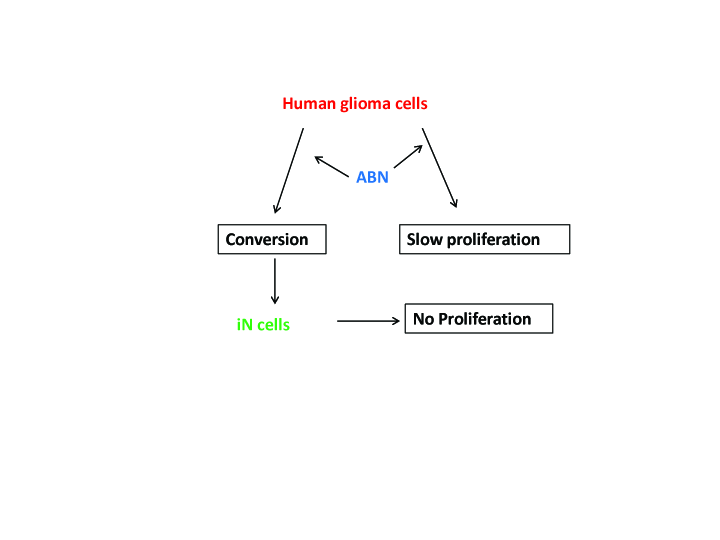

Supplement: Figure S5 — A model of the effect of ABN neuronal transcription factors on glioma cells conversion and proliferation. (TIF) [file pone.0041506.s005.tif]
